# The Language Used Around Diabetes: A Qualitative Study Focusing on the Experience of People Living With Type 1 and Type 2 Diabetes in Ireland

**DOI:** 10.1111/hex.70589

**Published:** 2026-02-08

**Authors:** Ellie Patterson, Méabh Finnegan, Sonya Deschênes, Michelle Lowry, Tomás P. Griffin, Ann‐Marie Creaven, Eimear C. Morrissey

**Affiliations:** ^1^ School of Psychology University of Galway Galway Ireland; ^2^ School of Psychology University College Dublin Dublin Ireland; ^3^ The Centre for Effective Services Dublin Ireland; ^4^ Centre for Endocrinology, Diabetes and Metabolism Galway University Hospitals Galway Ireland; ^5^ School of Medicine University of Galway Galway Ireland; ^6^ Department of Psychology University of Limerick Limerick Ireland; ^7^ Health Research Institute University of Limerick Limerick Ireland; ^8^ Centre for Health Research Methodology, School of Nursing and Midwifery University of Galway Galway Ireland; ^9^ Institute for Clinical Trials, College of Medicine, Nursing and Health Sciences University of Galway Galway Ireland

**Keywords:** communication, diabetes, Irish, language, qualitative, stigma

## Abstract

**Context:**

The majority of people living with diabetes experience stigma; this is often conveyed through language. There is a growing international focus on the language used around diabetes, but the experience of the same has not been investigated in an Irish context.

**Objectives:**

To investigate: (1) the experience of language related to diabetes among people living with type 1 or type 2 diabetes in Ireland and (2) perceptions of the Irish Language Matters guide.

**Methods:**

Using a descriptive qualitative approach, semi‐structured interviews were conducted with 14 participants aged 21–68, diagnosed with type 1 (*n* = 10; 8 female) or type 2 diabetes (*n* = 4; 2 female). Reflexive thematic analysis was used.

**Results:**

Four themes were developed: (1) ‘Language used by healthcare providers matters’; sub‐themes: ‘Dismissive, blameful, and inadequate language’ and ‘Person versus Patient’; (2) ‘Judgement’; sub‐themes: ‘Misunderstanding and misconceptions about diabetes’ and ‘Minimising and othering language’; (3) ‘More than language’; and (4) ‘The Irish Language Matters guide: Mixed feelings’. Language described was predominantly negative, emotionally meaningful, and important in healthcare. It often conveyed stigma, criticism and judgement rather than care and support. Communication as a whole, including tone and attitude, was important. The guide was largely endorsed, but disagreement existed around some terms.

**Conclusions:**

In Ireland, language around diabetes is typically negative and conveys stigma. This study underscores the need for Ireland‐specific guidance and training on respectful language and effective communication, particularly in healthcare settings.

**Patient or Public Contribution:**

The initial interview guide developed by the primary researcher was circulated to the Diabetes Language Matters Ireland Working Group, which includes people living with diabetes, psychologists, dieticians and a consultant endocrinologist, all Ireland‐based. Their feedback was incorporated across two rounds to produce the penultimate guide. This guide was then piloted by the primary researcher with a person living with type 1 diabetes; pacing adjustments were made to produce the final interview guide.

## 1 Introduction

Diabetes is a chronic condition wherein the pancreas makes insufficient insulin—the hormone that regulates blood glucose levels—or when the insulin produced by the body is no longer effectively used by cells [1]. Self‐care is critical in managing type 1 and type 2 diabetes (T1D and T2D, respectively). Depending on diagnosis, this involves monitoring blood glucose levels, injecting insulin, taking medication to compensate for suboptimal production and/or utilisation of insulin, and following specific food‐ and exercise‐related recommendations [2]—all daily. These management practices are numerous, representing a significant burden to people living with type 1 and type 2 diabetes (PLWD). This burden can be manifested in diabetes distress, encompassing feelings of helplessness, hopelessness, burnout and concerns about others' judgement [3].

Stigma represents a manifestation of labelling, stereotyping, separation, status loss and discrimination within a power system permitting them [4]. Blame, fear, disgust, enforcement of social norms, and disease avoidance are specific to diabetes stigma [5]. Experienced and perceived stigma is related not only to diabetes distress, but to more pronounced depressive symptoms, resulting in poorer quality of life, symptoms of anxiety and higher blood glucose levels over time [6, 7, 8, 9]. Moreover, the experience of stigma can result in disengagement from healthcare [10]. Most PLWD have experienced diabetes‐related stigma, which affects their emotional and social life and their diabetes management [11]. Many PLWD have reported feeling that diabetes is perceived as resulting from a character flaw or lack of personal responsibility [11]. Those who inject insulin have reported frustration at having to hide their self‐care to avoid others' discomfort, discrimination and looks of ‘contempt’ [12, p. 149]. As a result, PLWD report having delayed self‐management to avoid eliciting stigma [9, 12, 13]. Stigmatising attitudes towards PLWD are widespread, coming from the media, healthcare professionals (HCPs), and friends and family, with a sense of blame commonly described [14, 15]. Abdoli et al. [16] have demonstrated that PLWD have been stigmatised as being sick, weak or disabled, leading to a sense of otherness; as being a reminder of death; as being a drink or drug ‘abuser’; and as having a contagious disease. A commonly reported stigma is that diabetes is self‐inflicted due to an individual's choices [16].

Stigma can easily be conveyed through the language we use. Stigmatising language is common in diabetes care, affecting both how PLWD feel about themselves and how they experience diabetes. Themes found in such language included judgement and oversimplification and directives, with participants noting that they would prefer their HCPs to stop judging and labelling [17]. A lack of consensus exists regarding the experience and perception of stigma through language. For example, where some people living with T2D (plwT2D) have reported that an emphasis on lifestyle is positive in highlighting personal agency, others feel that it reinforces blameful attitudes, perpetuating stereotypes of plwT2D as lazy [14].

Diabetes stigma from any source negatively impacts emotional life, social life and self‐care [11]. However, particularly alarming is the prevalence of diabetes‐related stigma—and the language that conveys it—among HCPs. Per Goddu et al. [18], physicians' use of stigmatising language in medical records results in poorer attitudes towards those patients from subsequent physicians. Furthermore, stigmatising language (e.g., ‘cheat’ and ‘fails’) appears at a higher frequency in hospital admission notes for PLWD as compared to those with other conditions [19, p. 9]. Quality communication between patients and healthcare providers is essential to quality care provision and collaborative care, which itself supports a reduction in diabetes distress and improved self‐care and clinical outcomes among PLWD [20, 21]. Despite this, both physicians and PLWD note a reticence among the latter to discuss self‐care due to a fear of being judged and/or shamed [22]. To avoid perpetuating stigma through language, recommendations have been published, such as avoiding the word ‘control’ in reference to blood glucose levels; qualifying control as ‘good’ or ‘bad’, as often happens, imbues the message with a misplaced sense of morality [23].

Since Diabetes Australia published their initial ‘Language Matters’ (LM) position statement in 2011, there has been a growing focus on the language around diabetes, with other groups following suit. In early 2024, an Irish LM guide was published [24]. This document was developed by people living with diabetes, and care professionals and researchers in the field, all of whom were Ireland‐based. Content was guided by international research, as virtually no research currently exists detailing the experience of language by PLWD in Ireland. Culture and language are inextricably interwoven [25], and while existing research into the language around diabetes is illuminating, it cannot be entirely extrapolated to the Irish experience. As demonstrated, language is significant in conveying stigmatising attitudes towards diabetes, moderating diabetes distress and promoting collaborative care for PLWD, yet no qualitative study explores the experiences of PLWD in Ireland as regards language. This represents a significant gap in our understanding of the above; the present study aims to begin addressing this gap.

## 2 Materials and Methods

### 2.1 Design

This was a qualitative study employing semi‐structured interviews, guided by a descriptive qualitative approach, per Sandelowski [26, 27], wherein the aim is to describe and summarise participants' experiences, interpreting the data while staying close to them. With the experiences of PLWD fundamental to this research, this approach facilitated a straightforward description of the same. The study was reported using the COREQ checklist [28]. All study materials are available at https://osf.io/wfy4c/.

### 2.2 Sample

Purposeful sampling [29] was used to ensure variation in age, gender and diagnosis. These characteristics were selected to guide purposive variation, and we did not use a formal sampling matrix given the focused nature of this sampling approach. Eligible participants were currently living—or had recently lived—in Ireland, were over 18 years of age, and had a diagnosis of either T1D or T2D. Data saturation, per Braun and Clarke [30], is incongruent with the principles of reflexive thematic analysis; as such, ‘information power’, per Malterud et al. [31], guided the sample size, along with available resources. Per information power, ‘the more [relevant] information the sample holds, the lower number of participants is needed’, with ‘the adequacy of the final sample size […] evaluated continuously during the research process’ (p. 1759). Participants offered insight into an unresearched area (in Ireland), and the quality of dialogue was reinforced by piloting the interview guide and by assessment of the quality of each interview upon its conclusion to address weaknesses. This enhances information power on the dimensions of aim, specificity and dialogue. Similar research by Browne et al. [15] and Browne et al. [14] comprised samples of 27 and 25, respectively; given the narrower focus of this research and the comparatively limited resources available, a final sample of 14 participants was deemed acceptable.

### 2.3 Recruitment

A flyer, which advertised the research and invited eligible participants, was developed by the researcher and posted in the online support group ‘Diabetes in Ireland’, which is hosted on Facebook, placed in the waiting room and toilet cubicles of the diabetes clinic of University Hospital Galway, posted on X by @healthpsygalway, circulated in the ‘Thriveabetes’ (T1D support group) monthly newsletter, and shared within the networks of members of the Diabetes Language Matters Ireland Working Group (DLMIWG). Two book tokens, each worth €50.00, were advertised on the flyer as items to be raffled amongst participants.

### 2.4 PPI

Patient and Public Involvement (PPI) was drawn on in developing the interview guide to elicit the richest and most relevant data. The primary researcher (E.P.) developed an initial guide based on that used by Dickinson [17], but expanded to better address the research question. This was then circulated to the DLMIWG, which includes PLWD, psychologists, dieticians and a consultant endocrinologist, all Ireland‐based. Their feedback was incorporated to produce the penultimate guide. This guide was then piloted by E.P. with a person living with T1D (plwT1D); adjustments were incorporated to produce the final interview guide. A document outlining the full interview guide development process can be seen at https://osf.io/wfy4c/files.

This guide encompassed various questions relating to participants' experience of language around diabetes—and diabetes‐specific language—in various contexts.

### 2.5 Procedure

After written informed consent was obtained, demographic questionnaires were distributed to each participant. Both forms were hosted by Microsoft Forms under the licence of University of Galway. Two participants did not complete the demographics questionnaire. One‐on‐one interviews were then conducted by E.P. with participants over Zoom, also under the licence of University of Galway. There was no relationship between E.P. and any participant prior to interview. In addition to the written informed consent, oral consent was obtained at the commencement of each interview. Interviews lasted between 31 and 110 min (*M* = 54 min, *SD* = 20.82 min). Audio and video were recorded; the video component was deleted upon each interview's conclusion. The interview guide provided the direction for each interview. The same base questions were asked of all participants, with different follow‐ups based on responses. The Irish LM guide was shared with each participant to prompt discussion about the same. This is a concise, 6‐page guide designed for anyone who writes or speaks about diabetes. This was shared at the end of each interview so as not to elicit any bias towards interview questions. Participants were asked whether they would prefer to read the guide in its entirety and then discuss, or go through the guide page‐by‐page and discuss throughout. Participants were reassured that they could review the guide at their own pace. No field notes were made during interviews. Interviews were recorded to the University of Galway Cloud using Zoom, and transcripts were obtained from Zoom. Once transcripts were edited and finalised, the audio recordings were also deleted. Three of the interviews were transcribed using Zoom alone; 11 were transcribed using Otter.ai, as this was later found to be more efficient. All data management and storage were compliant with GDPR. Transcripts and findings were not returned to participants for comment, correction or feedback. A debrief was issued to participants.

### 2.6 Analysis

Data were analysed inductively using reflexive thematic analysis, per Braun and Clarke [32, 33]. The six steps prescribed were adhered to: familiarisation with data (facilitated by transcription editing and re‐reading), generation of initial codes and generation of themes using NVivo [34], review of themes, definition and naming of themes, and finally, production of the report. E.P. coded the data and reviewed the themes. E.M. (a female health psychologist and supervisor of E.P.) served as a ‘critical friend,’ providing peer debriefing by challenging and developing E.P.'s interpretations. Themes were defined following discussion with E.M. Coding was executed using NVivo 20 [34].

### 2.7 Ethics

Regarding confidentiality, with in‐depth one‐on‐one interviews came the possibility that, from direct quotes implemented in the results, someone could be identified based on specific instances relayed. Informed consent for the use of such quotes was obtained from participants. Aside from this, data were pseudonymised; identifying information, such as clinic locations, was removed from transcripts. Once transcripts were finalised, only the transcripts themselves and anonymous demographic data remained and were held on the University of Galway OneDrive system. Interview questions were designed to prompt participants to speak at length about their diabetes, which is a very personal health experience. There was potential for embarrassment, discomfort and distress within these interviews. In order to mitigate this as best as possible, a distress protocol was developed should this arise; ultimately, this was not required during any interview. Prior to recruitment and data collection, ethical approval for this research was sought and obtained from the University of Galway School of Psychology Research Ethics Committee.

## 3 Results

**Figure 1:**
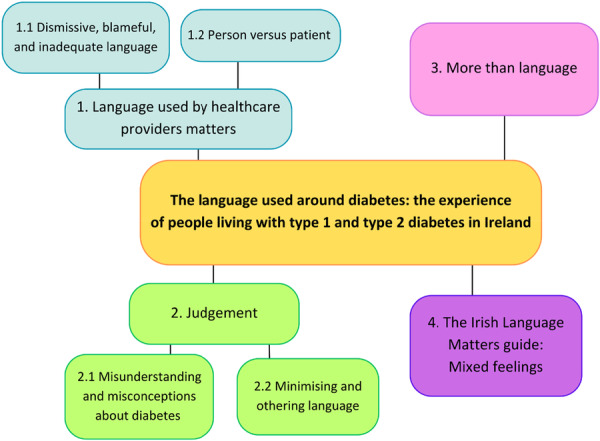
Results of analysis: Themes and sub‐themes.

14 participants were interviewed; their characteristics are displayed in Table 1. None withdrew during or post interview. Using reflexive thematic analysis, the researcher generated four themes from the data (see Figure 1): ‘Language from HCPs matters’, ‘Judgement’, ‘More than language’ and ‘Irish Language Matters document’, which presents an organisation of participants' responses to the same.

**Table 1:** Participant demographics.

Participant code	Gender	Age (years)	Diagnosis
P01	Female	—	T1D
P02	Female	27	T1D
P03	Female	23	T1D
P04	Male	51	T1D
P05	Male	33	T1D
P06	Female	28	T1D
P07	Female	45	T1D
P08	Female	46	T2D
P09	Male	68	T2D
P10	Female	64	T2D
P11	Male	—	T2D
P12	Female	51	T1D
P13	Female	40	T1D
P14	Female	21	T1D

### 3.1 Theme 1: Language From HCPs Matters

Language used by HCPs was consistently represented as impactful, with much of this language experienced negatively. This theme encompasses the sub‐themes ‘Dismissive, blameful, and inadequate language’ and ‘Person versus patient’.

#### 3.1.1 Sub‐Theme 1.1: Dismissive, Blameful and Inadequate Language

Participants experienced dismissive language from HCPs, which commonly conveyed a sense that participants' diagnoses were theirs alone to manage. Participants reported being told by HCPs that ‘you're an intelligent lady […] you'll work it out’ (P01), ‘it's up to yourself’ (P09) and to ‘figure it out’ (P05). This was commonly experienced at or early in diagnosis. While this approach was not experienced negatively by every participant (‘it sort of woke me up’ [P09]), it was typically described in negative terms (‘[i]t was just the most frustrating thing’ [P12]) and led participants to feel that ‘this whole thing is my responsibility’ (P09). Participants reported that language from HCPs was sometimes insufficient in communicating important health concepts, which were ‘washed over’ (P06). Vague language that downplayed the seriousness of diabetes was also relayed, which could lead people at risk of developing T2D to take diabetes less ‘seriously’ (P03) than they otherwise might have: ‘for years and years, I was told, “Oh, you're on the road to diabetes, you're on the road to diabetes,” […] So what? Like, what does that mean?’ (P08).

This sense of dismissal often intersected with blameful, critical language; P01 was ejected from an appointment with her consultant (‘Get out!’), being told that she wasn't ‘taking this seriously’. One HCP, because he couldn't explain P10's symptoms, asked her whether she was ‘making them up’, which ‘really, really floored me and really upset me…. And actually still does’. Some HCPs were reported as being relentlessly critical without offering help or explanation, using phrases like ‘not very good at all […] you're doing it wrong’ (P05). Furthermore, serious complications were seemingly raised with participants as criticisms rather than legitimate health concerns; P12 was told by her consultant that her baby ‘would be born with either anencephaly or encephalitis’ because her bloods weren't ‘controlled’ at conception, which ‘ruined the whole pregnancy’. P14 was told by an HCP that she would ‘die in [her] sleep’ after her insulin pump failed.

This language negatively affected participants, who reported being ‘terrified’ (P01; P07) in anticipation of appointments, feeling ‘I'm doing it wrong’ (P07). Participants described consequently wanting to withhold health data, and even to avoid healthcare, explaining that ‘it would have brought me to, kind of, tears […] from the age of 18 to the age of 24, 25, I stopped going to my clinic…’ (P05). Participants preferred when HCPs used neutral language or offered praise (‘[y]ou're doing really well’ [P14]), support (‘is there anything I can do?’ [P13]) and reassurance (‘it will be all right, we'll watch the sugars…’ [P12]) instead of blame, explaining that ‘[y]ou're willing to open up because you don't feel like you're going to be attacked’ (P05). However, such instances were not common: ‘I remember a nurse saying, “it's not your fault,” which is the only positive thing I've ever heard about diabetes in my life—that's it’ (P08).

#### 3.1.2 Sub‐Theme 1.2: Person Versus Patient

Language from HCPs was often described as either acknowledging or denying the personhood of PLWD and the fact that they had full, complex lives outside of their diagnoses. The former was described as supportive; the latter was described as having ‘no sensitivity to it, and no humanity’ (P05).

P07, for example, reported: ‘I was diagnosed when I was 17 and I'm 45 now, and only *once* have I ever been asked how I was actually feeling’. Communicating with HCPs was described as being ‘like a script’ (P05) and ‘box‐ticking’ (P07); ‘[t]hat's a script that they're reading from’ (P08). This was particularly frustrating as, per P05, ‘diabetes is personal’.

An approach centring data in neutral terms was received positively and allowed for some ‘distance’ (P09) between participants and their diagnoses. However, many participants preferred that medical data and figures, while important, were secondary to the overall experience of PLWD when discussing diabetes: ‘[i]t wasn't “That's low. That's wrong.” It was, “That's low […] is it having an effect on you?”' (P12). When figures were the only thing spoken about, participants reported feeling a lack of caring from HCPs regarding their diabetes:

I've always come out of the breast clinic feeling, you know, that was […] very caring. And I've never come out of my GP with regards to diabetes feeling ‘that was caring’ […] He never really asks me how I'm feeling […] [H]e just takes the blood, and then, you know, he, he'll take my blood pressure, and, so, he'll run through the checks […] It's just fact‐giving. It's not, there's no sense of, really, care.

P10

Many participants valued simply being asked how they were doing; it signalled that their HCP cared about them: ‘[h]e wants to know, you know, about the emotions. “How has it been? What's been going on, em, what has been impacted?”’ (P01). The words ‘control’ and ‘compliance’ were experienced as impersonal and dehumanising: ‘to fit into a box is very difficult. […] [W]hat am I complying to? Like, somebody's version of what […] “good” numbers are’ (P07); ‘you control prisoners, you control cattle, you control *things*, and it's just, it's, it doesn't feel human almost’ (P05).

### 3.2 Theme 2: Judgement

The theme ‘Judgement’ was generated across the data. Participants experienced judgemental language from HCPs, family, friends, strangers and colleagues. From HCPs, self‐management was often the target of such language, with terms like ‘bad’, ‘good’ and ‘control’ used to describe blood glucose levels, instead of more objective—and preferable—terms like ‘high’ and ‘low’, or simply providing a figure: ‘referring to, like, “bad” results, or “bad” blood sugars […] like, a blood sugar result is just a data point’ (P06). The term ‘control’ was encountered both from HCPs and non‐HCPs; participants felt that this term implied judgement of effort, not acknowledging that PLWD are ‘all doing [their] best’ (P07), and that self‐management is complex: ‘control implies that, there, you *can* control it…. And you can't’ (P12). HCPs' persistent questioning of care needs and preferences conveyed judgement of participants' expertise: ‘[t]hey'll keep asking why; “Why do you want [a different sensor]?” Like, “prove it to me, give me a presentation, prove it to me with your blood sugar results”’ (P03). HCPs referred to the ‘effort’ they judged PLWD to be making, implying that this would affect their care. P14 recalled, from childhood, being told that ‘[t]here's other kids who would make more of an effort than you, so we might give [your pump] to them, instead’.

Participants reported repeatedly hearing judgements or ‘diktats’ (P09) from friends, family, colleagues, and strangers involving what they ‘could’, ‘couldn't’, ‘mustn't’, ‘can't’ and are ‘not allowed’ to do. Participants noted that if diabetes had to be raised, they would be ‘receptive’ to words of support and broad questions like ‘well done,’ or ‘how's it going?’, which were ‘encouraging’ (P09). Judgemental language was often interwoven with misconceptions and language that minimised PLWDs' experience and highlighted them as different; as such, two sub‐themes within the theme of ‘Judgement’ are ‘Misunderstanding and misconceptions about diabetes’ and ‘Minimising and othering language’.

#### 3.2.1 Sub‐Theme 2.1: Misunderstanding and Misconceptions about Diabetes

Participants faced judgemental and tactless comments based on misconceptions. P01 reported hearing ‘[y]ou don't *look* like a diabetic. My uncle is really fat, and you don't seem to have that *fat* diabetes thing’. ‘Sugar’, and consequently overall health, was consistently misunderstood by people who didn't live with diabetes; ‘[o]h, did you eat too much sugar when you were a kid?’ (P07). P13 reported a friendship ending due to the other party telling her ‘that if I ate a vegan diet I would be able to reverse my diabetes. […] The complete and total ignorance’. Many noted these comments as frequent: ‘[i]t's happening all the time to somebody, maybe not always me, but to somebody’ (P07). Misconceptions were typically attributed to the general public, although P02 reported being asked by an HCP whether her T1D was ‘cured’, which she described as ‘irritating’ and signalling a ‘lack of awareness’. Participants often did not oppose being asked ‘polite’ and carefully worded questions around diabetes, and some viewed this as positive, if only because having received their answer, the asker would not have to question another PLWD. All the same, repeatedly correcting misconceptions was ‘frustrating’ and ‘tedious’ (P05); it reminded participants of the challenges of diabetes (‘the fact that you can't do that floods into my brain’ [P05]) and gave a sense that ‘privacy’ (P03) was lost or compromised.

The word ‘diabetes’ itself was noted as being unclear and undescriptive, and thus engendering misconception. Participants remarked that T1D and T2D are vastly different and that the umbrella term of ‘diabetes’ perpetuates confusion between them. P08 reported that it was ‘embarrassing’ to refer to her T2D as ‘diabetes’ when speaking with a friend with T1D, because hers is ‘somewhat self‐inflicted’, demonstrating what could be described as an internalised judgement of her own T2D. Participants frequently encountered judgemental comments relating to types of diabetes. T1D was alternately referred to as the ‘bad’ kind (owing to the presumed involvement of injections) and the ‘good’ kind (owing to ideas about lifestyle): ‘they go, “Oh, you don't have *that* one then that, like, […] you're kind of healthy”’ (P02); ‘“[o]h, you've got the *bad* diabetes,” and you're just like, what does that mean to you? Um, and why do you need say that?”’ (P06). Participants, both with T1D and T2D, expressed that when discussing ‘diabetes’, the type of diabetes should always be clarified.

#### 3.2.2 Sub‐Theme 2.2: Minimising and Othering Language

Participants often noted language that singled them out and minimised their experience. This is exemplified through ‘jokes’ relating to diabetes: ‘“Oh, look at your woman, you know, taking drugs,” or […] just making a joke out of it’ (P07). These ‘jokes’ sometimes involved labels, such as, per P05, ‘The Diabetic’ or ‘The Cyborg’, as used by his manager; ‘it's a joke, but it's not really a joke […] it kind of felt, em, like you're being looked at like you've got a disability—like you're different’. Jokes invoked stereotypes and had an offhand, naïve tone, for example, pictures of food captioned ‘hashtag diabetes, lol!’ (P03), ‘diabetes on a plate’ (P12), and that an Easter egg was ‘gonna give me diabetes’ (P06). These jokes ‘annoy[ed]’ (P03) participants, some of whom took them ‘very personally’ (P10).

Other minimising comments had an overtly judgemental tone; participants reported being told that other PLWD don't ‘go on about it as much as you do’ (P01), and being asked ‘[w]hy are you making it such a big deal?’ (P03). Conversely, participants noted that people who did not have diabetes often used language to highlight it; P03 reported discovering that she was referred to as ‘Diabetes Girl’, which ‘frustrated’ and ‘hurt’ her; P13 recalled hearing a neighbour say to her father, ‘oh, this is your little sick child’. P10 described an instance in a café where a friend took an item from her plate and returned it to the waitress, announcing: ‘She can't have that, she's diabetic’. Despite being ‘really mortified’, P10 felt she couldn't address the situation because doing so would draw more unwanted attention. For some, the term ‘diabetic’ itself was othering; it was recommended that ‘the right language, I suppose, is […] not to see people who have diabetes as *diabetics*, you know, but they're people who have diabetes’ (P10). Although for others, the term ‘diabetic’ was not inherently loaded: ‘[d]on't take one [term] and then use it as a negative. […] I'm not a person with diabetes. I am a diabetic’ (P04).

Judgemental and othering language was used where participants were asked or commanded to hide their self‐care, particularly around injecting. P01 recalled being told ‘you don't need to be showing us every time’, ‘just go away’ and ‘go into the toilet and do it’, remarking that ‘you just get very, very, very upset, *very* upset’. P12, who provided care for a young person also living with T1D, was asked by a colleague ‘would it not be more hygienic and more dignity to go down to the bathroom to do that?’ P12 felt ‘[c]ompletely disrespected’.

Participants appreciated language acknowledging, rather than minimising, the challenges of diabetes. P05 recalled, positively, a woman praising his injecting insulin in public, saying that it was ‘impressive’ and that he ‘should be so proud’. P02 recalled a former manager calling her after having developed gestational diabetes, apologising for not having ‘realise[d] the challenge of it’ before: ‘“I didn't know the struggle of it until, am, I was diagnosed.” […] I'll always remember that experience in a positive way…’. However, overemphasis of the ‘struggle’ of diabetes could read as patronising or even ‘pity[ing]’ (P05); ‘it's like, you know, here's a chocolate biscuit or a gold star. […] [S]he'll kind of say “Oh sure, I know, it's so hard.” Don't tell me it's hard, because it's not’ (P13).

### 3.3 Theme 3: More Than Language

A theme clear in these data is that being spoken to and communicated with about diabetes encompasses more than words and language alone. Most participants noted that attitude, tone, context, intent and one's own perception matter in processing language. Participants reported emotionally evocative experiences involving language throughout these interviews, and sometimes specific words could not be recalled: ‘I can't remember what they were, but they were jarring’ (P01).

Sometimes it was not the words themselves that were evocative, but the manner in which they were delivered: ‘she'd probably get a bit quiet, or she'd kind of whisper it, or be like [whispered]: “She has diabetes.” Em, ah, so it's not that she says words that are particularly negative, but it's more the tone…’ (P02); ‘it's not what you say, it's the way you say it’ (P11).

Participants reported that the intent they perceive from the speaker informs their view of what is said and that the same words from two different people could have different effects: ‘it's the intention of how you approach it, like this guy was very apprehensive, and he didn't want to say the wrong thing’ (P06); ‘it was his truthfulness, honesty, and because he has integrity, I kind of took it on board, and I didn't get insulted about it’ (P09). While language and words are impactful, the effect of nonverbal communication and perceived intent cannot be disregarded.

### 3.4 Theme 4: Irish LM Guide: Mixed Feelings

Participants were shown the Irish LM document and asked for their opinion on it. Most participants approved of the document, remarking that it was ‘a great resource’ (P02) and that ‘[i]t's really informative and useful’ (P07). Many identified with the quotes (p. 4) and the discussion of how words are impactful (p. 3), commenting that they ‘ring very true’ (P10), and that they accurately reflected many interview topics.

The LM document stimulated discussion on specific words and phrases. No participant argued that the use of the words ‘sufferer’ and ‘normal’ (when describing people not living with diabetes) was acceptable, for the reasons cited in the document. Certain terms prompted more mixed views, in particular, ‘blood sugar’, ‘diabetic’ and ‘control’.

More participants were comfortable with the term ‘blood sugar’—as opposed to the recommended ‘blood glucose’—than opposed it. Many participants were simply ‘fine’ with either term. Reasons for preferring ‘sugar level’ were that it made communication ‘easier’ (P12), especially when talking to those who do not have diabetes, and that sugar was ‘a less scary word than “glucose”’ (P14). Those who preferred ‘glucose’ were in the minority, noting that saying ‘sugar’ contributed to the misconception that diabetes is caused by, or revolves around, foods that taste sweet or contain lots of refined sugar. As discussed earlier, some participants agreed that the term ‘diabetic’ shouldn't be used as it indicates that diabetes is one's ‘identity’ (P02). Other participants were either indifferent or felt that ‘diabetic’ was a neutral, descriptive term that one doesn't ‘have to take […] as a definition of yourself’ (P04); some who were indifferent noted that using ‘person with diabetes’ was inoffensive and ought to be the default if uncertain. Some perceived the term control as neutral and ‘appropriate in certain occasions’ (P04), remarking that ‘“well‐controlled” is a good descriptive indicator of how you're managing your glucose levels’ (P08).

Notably, P08 objected to the document on the grounds that it was essentially ‘telling’ PLWD what words they should and shouldn't use. P04 wondered whether we're not ‘reading too much into these words’, and P11 reported that it focused too much on ‘negativity’ and wouldn't ‘motivate’ him because it takes the ‘responsibility or the accountability away from the patient’.

## 4 Discussion

The objective of this study was to explore the experience of language in the context of T1D and T2D in Ireland. Four themes were generated. ‘Language from HCPs matters’ encompassed the sub‐themes ‘Dismissive, blameful, and inadequate language’ and ‘Person versus patient’. The theme ‘Judgement’ encompassed the sub‐themes ‘Misunderstanding and misconceptions about diabetes’ and ‘Minimising and othering language’. The final two themes were ‘More than language’ and ‘Irish Language Matters document’; these had no sub‐themes. Broadly speaking, findings echo work by Dunning et al. [35], demonstrating that language around diabetes is predominantly negative, encompassing many words that are judgemental or moralising. Such words and phrases have a strong affective impact on PLWD [36], resounding with present findings. Moreover, much of the language described by participants conveyed diabetes stigma as described by Speight et al. [10] and Schabert et al. [5], like stereotypes and negative social judgements.

Findings align with vast research demonstrating that the language used by HCPs is impactful (e.g., [17, 37, 38, 39]). HCPs' language was often experienced as judgemental, blameful, dismissive, and as denying personhood; consequently, participants reported distress, fear and frustration, in line with research by Dickinson [17]. Resultant healthcare avoidance—in one case for several years—was described. Stigma affects healthcare engagement [10]; present findings tentatively suggest that language could mediate this. Many PLWD wish to speak with their HCPs about their ‘feelings and personal experience of living with diabetes’ [40, p. 475] and want their HCP to recognise that after a certain time, they, PLWD, are the expert in their diabetes [41]; present findings reflect this. PLWD associate better communication with empathy, compassion and explanation of conditions, with fear of judgement deterring their disclosure of health information [42, p. 14]. Correspondingly, participants herein preferred neutral or supportive language which centred their overall experience and avoided judgemental words, like ‘bad’, when referring to health data.

Language typically conveyed judgement, via misconceptions about diabetes, and minimising and othering language. The term ‘control’ was frequently mentioned and objected to as judgemental and dehumanising, being encountered often, and from various sources. ‘Control’ has been widely demarcated as inappropriate, for example, by Cooper et al. [37]. Minimising, othering language related by participants sometimes had a flippant quality, which, like that cited by Blackwood et al. [43], often did not seem intended to admonish PLWD. This may be because many people do not view diabetes as stigmatised (Schabert 2013), and thus may not appreciate that their words perpetuate stigma. Dismissive language appeared in ‘jokes’ about diabetes, an area rarely discussed in LM disseminations. Other language was more overtly judgemental, carrying commands and directives despite speakers often misunderstanding diabetes and failing to appreciate the demands involved in self‐management—a common phenomenon [44, 45]. Overall, findings align with current research describing diabetes stigma [10] and suggest that language is impactful in conveying this stigma.

It was widely reported that while words matter, the tone and manner in which they are expressed are significant; the theme ‘More than language’ communicates this. Much research on the language around diabetes comments on specific words; in the present study, only some individual words, for example, ‘control’, appeared repeatedly. Often, words that could otherwise have been neutral were transformed by their delivery or intent. This could be cultural: Barron [46] submits that ‘avoiding forwardness in language use’ represents a ‘socio‐cultural [value] in Irish society’ (p. 403), thought to conserve ‘ambiguity and neutrality of opinions’ and avoid confrontation [47, p. 20] [48]. Likewise, an Irish linguistic approach can favour ‘indirectness’, encompassing various nonverbal idiosyncrasies [49, p. 457], with what is unsaid being equally (or more) important than what is said. It has been noted that, in Ireland, directness is more acceptable through humour, which ‘can be use[d] as a tool to express criticism’ [50, p. 157]. This highlights the need for distinct guidelines vis‐à‐vis communication as a whole around diabetes in an Irish context. These guidelines may diverge from those set out in the United Kingdom, which only briefly mention ‘verbal and non‐verbal’ language [51, p. 5]. In 2019, the HSE launched the National Healthcare Communication Programme for HCPs, which has a module on nonverbal communication; this could be adapted into guidelines for communicating with and about PLWD.

Most participants had a positive view of the Irish LM document, largely agreeing with the proposed recommendations. Curiously, many participants approved of the term ‘blood sugar’ as opposed to the recommended ‘blood glucose’, even though ‘sugar’ was often cited during interviews as a word associated with misconception and judgement. PLWD often ‘[take] on the labor of educating […] others’ [45, p. 52]; using a less colloquial term like ‘glucose’ may prompt unwanted questions from those unfamiliar with diabetes. As demonstrated both via the interview questions and the discussion on the Irish LM document, opinions on the term ‘diabetic’ varied, echoing current debate over person‐first (e.g., ‘person with diabetes’) versus identity‐first (e.g., ‘diabetic’) language (see [52]). While the former promotes personalised language, avoiding terms like ‘diabetic’ could create a sense of taboo and actually emphasise stigma [53]. The aim of person‐first language is inclusion, yet such language is often used to refer to those with the *most* stigmatised conditions [54]. Current findings suggest that the most appropriate language is that preferred by PLWD, but that person‐first language is most prudent if unsure [24].

The present study was subject to limitations, such as self‐selection bias, as observed by Costigan and Cox [55]: participants volunteered to be interviewed, meaning some may have been motivated to speak about striking experiences with diabetes‐related language, which are not necessarily representative. Women were over‐represented in this study, as is often seen in research calling for self‐disclosure [56]. PlwT1D were also in the majority; research suggests that, in some cases, plwT1D may be affected more negatively by ‘negative’ terms than plwT2D [36], although this may be answerable to self‐stigmatisation among plwT2D (Browne et al., 2016, as cited in [36]), that is, plwT2D may view as objective, or agree with, terms viewed as negative by plwT1D. As interviews and study materials were conducted in English and required reading a brief written document, individuals with lower literacy levels or for whom English is not a first language may have been unintentionally excluded, which we acknowledge as a limitation of the study.

Based on findings, future research could determine whether the experience of language, specifically, quantifiably affects healthcare engagement, and whether this experience is mediated by self‐stigmatisation. Current findings have predominantly described language that is experienced negatively. Although, as discussed, language around diabetes does appear to be largely negative, future research could explore positive language in a focused way. Future research might also explore Ireland‐based HCPs' views on language around diabetes, which, in combination with present findings, might help to implement best practice. Based on findings, there may be a need for guidance for HCPs specifically around language and communication. The current Irish LM guidance appears apt, but could discuss nonverbal communication and diabetes ‘jokes’ to a greater extent.

## 5 Conclusions

This is the first qualitative study investigating the experience of language around diabetes by PLWD in Ireland. It was found that much of this language is negative, conveying judgement via misunderstanding, othering and minimising. Language used by HCPs could be inadequate and was impactful in conveying dismissal and blame as opposed to care and support, and in conveying HCPs' concept of PLWD as patients versus as people. Participants reported that such language had a negative emotional effect and affected their engagement with healthcare. Divergence of opinion was observed regarding certain terms, such as ‘diabetic’, and the philosophy behind them. Beyond language, communication as a whole, including tone, attitude and intent, was described as important. The Irish LM document was largely endorsed, with a minority of participants finding it overly prescriptive or unwarranted.

## Author Contributions

**Ellie Patterson:** data curation, investigation, methodology, project administration, visualisation, writing – original draft, writing – review and editing. **Eimear Morrissey:** conceptualisation, methodology, project administration, resources, supervision, writing – review and editing. **Méabh Finnegan:** methodology, writing – review and editing. **Sonya Deschênes:** conceptualisation, methodology, writing – review and editing. **Michelle Lowry:** conceptualisation, methodology, writing – review and editing. **Tomás P. Griffin:** conceptualisation, methodology, writing – review and editing. **Ann‐Marie Creaven:** conceptualisation, methodology, writing – review and editing.

## Funding

The authors received no specific funding for this work.

## Ethics Statement

Ethical approval for this research was sought and obtained from the University of Galway School of Psychology Research Ethics Committee.

## Conflicts of Interest

The authors declare no conflicts of interest.

## Data Availability

The materials and datasets generated and/or analysed during the current study are available in the OSF repository.

## References

[1] World Health Organisation , April 5, (2023). *Diabetes*, https://www.who.int/news-room/fact-sheets/detail/diabetes#:~:text=Overview,hormone%20that%20regulates%20blood%20glucose.

[2] Diabetes Ireland , n.d. *Living With Diabetes*, https://www.diabetes.ie/living-with-diabetes/.

[3] L. Fisher , W. H. Polonsky , and D. Hessler , “Addressing Diabetes Distress in Clinical Care: A Practical Guide,” Diabetic Medicine 36, no. 7 (2019): 803–812, 10.1111/dme.13967. 30985025

[4] B. G. Link and J. C. Phelan , “Conceptualizing Stigma,” Annual Review of Sociology 27, no. 1 (2001): 363–385, 10.1146/annurev.soc.27.1.363.

[5] J. Schabert , J. L. Browne , K. Mosely , and J. Speight , “Social Stigma in Diabetes: A Framework to Understand a Growing Problem for an Increasing Epidemic,” Patient—Patient‐Centered Outcomes Research 6 (2013): 1–10, 10.1007/s40271-012-0001-0. 23322536

[6] S. Akyirem , E. Ekpor , D. Namumbejja Abwoye , J. Batten , and L. E. Nelson , “Type 2 Diabetes Stigma and Its Association With Clinical, Psychological, and Behavioral Outcomes: A Systematic Review and Meta‐Analysis,” Diabetes Research and Clinical Practice 202 (2023): 110774, 10.1016/j.diabres.2023.110774. 37307898

[7] D. Gredig and A. Bartelsen‐Raemy , “Diabetes‐Related Stigma Affects the Quality of Life of People Living With Diabetes Mellitus in Switzerland: Implications for Healthcare Providers,” Health & Social Care in the Community 25, no. 5 (2017): 1620–1633, 10.1111/hsc.12376. 27489251

[8] U. M. Hansen , K. Olesen , and I. Willaing , “Diabetes Stigma and Its Association With Diabetes Outcomes: A Cross‐Sectional Study of Adults With Type 1 Diabetes,” Scandinavian Journal of Public Health 48 (2020): 140349481986294, 10.1177/1403494819862941. 32338563

[9] R. Kaur and A. Sinha , “Perceived Stigma Among Diabetic Patients and Their Caregivers: A Review,” Perspectives in Public Health 144, no. 4 (2024): 242–250, 10.1177/17579139221136725. 36633308

[10] J. Speight , E. Holmes‐Truscott , M. Garza , et al., “Bringing an End to Diabetes Stigma and Discrimination: An International Consensus Statement on Evidence and Recommendations,” Lancet Diabetes & Endocrinology 12 (2024): 61–82, 10.1016/S2213-8587(23)00347-9. 38128969

[11] N. F. Liu , A. S. Brown , A. E. Folias , et al., “Stigma in People With Type 1 or Type 2 Diabetes,” Clinical Diabetes 35, no. 1 (2017): 27–34, 10.2337/cd16-0020. 28144043 PMC5241772

[12] A. Tak‐Ying Shiu , J. J. Y. M. Kwan , and R. Y. M. Wong , “Social Stigma as a Barrier to Diabetes Self‐Management: Implications for Multi‐Level Interventions,” Journal of Clinical Nursing 12, no. 1 (2003): 149–150, 10.1046/j.1365-2702.2003.00735.x. 12519263

[13] J. A. Nettleton , A. E. Burton , and R. C. Povey , “‘No‐One Realises What We Go Through as Type 1s’: A Qualitative Photo‐Elicitation Study on Coping With Diabetes,” Diabetes Research and Clinical Practice 187 (2022): 109876, 10.1016/j.diabres.2022.109876. 35439539

[14] J. L. Browne , A. Ventura , K. Mosely , and J. Speight , “‘I Call It the Blame and Shame Disease’: A Qualitative Study About Perceptions of Social Stigma Surrounding Type 2 Diabetes,” BMJ Open 3, no. 11 (2013): e003384, 10.1136/bmjopen-2013-003384. PMC3840338 24247325

[15] J. L. Browne , A. Ventura , K. Mosely , and J. Speight , “‘I'm Not a Druggie, I'm Just a Diabetic’: A Qualitative Study of Stigma From the Perspective of Adults With Type 1 Diabetes,” BMJ Open 4, no. 7 (2014): e005625, 10.1136/bmjopen-2014-005625. PMC4120421 25056982

[16] S. Abdoli , M. Doosti Irani , L. R. Hardy , and M. Funnell , “A Discussion Paper on Stigmatizing Features of Diabetes,” Nursing Open 5, no. 2 (2018): 113–119, 10.1002/nop2.112. 29599986 PMC5867293

[17] J. K. Dickinson , “The Experience of Diabetes‐Related Language in Diabetes Care,” Diabetes Spectrum 31, no. 1 (2018): 58–64, 10.2337/ds16-0082. 29456427 PMC5813309

[18] A. P. Goddu , K. J. O'Conor , S. Lanzkron , et al., “Do Words Matter? Stigmatizing Language and the Transmission of Bias in the Medical Record,” Journal of General Internal Medicine 33 (2018): 685–691, 10.1007/s11606-017-4289-2. 29374357 PMC5910343

[19] G. Himmelstein , D. Bates , and L. Zhou , “Examination of Stigmatizing Language in the Electronic Health Record,” JAMA Network Open 5, no. 1 (2022): e2144967, 10.1001/jamanetworkopen.2021.44967. 35084481 PMC8796019

[20] J. K. Dickinson , R. E. Posesorski , S. G. Djiovanis , and V. J. Brady , “Impact of Negative or Stigmatizing Messages on Diabetes Outcomes: An Integrative Review,” The Science of Diabetes Self‐Management and Care 50, no. 2 (2024): 167–178, 10.1177/26350106241232644. 38454649

[21] T. C. Skinner , L. Joensen , and T. Parkin , “Twenty‐Five Years of Diabetes Distress Research,” Diabetic Medicine 37, no. 3 (2020): 393–400, 10.1111/dme.14157. 31638279

[22] M. D. Ritholz , E. A. Beverly , K. M. Brooks , M. J. Abrahamson , and K. Weinger , “Barriers and Facilitators to Self‐Care Communication During Medical Appointments in the United States for Adults With Type 2 Diabetes,” Chronic Illness 10, no. 4 (2014): 303–313, 10.1177/1742395314525647. 24567195 PMC4157962

[23] J. Speight , J. Conn , T. Dunning , and T. C. Skinner , “Diabetes Australia Position Statement. A New Language for Diabetes: Improving Communications With and About People With Diabetes,” Diabetes Research and Clinical Practice 97, no. 3 (2012): 425–431, 10.1016/j.diabres.2012.03.015. 22513346

[24] S. Birney , C. Breen , A.‐M. Creaven , et al., 2024. *Talking About Diabetes: Language Matters*. Diabetes Ireland, https://www.diabetes.ie/wp-content/uploads/2024/02/talking-about-diabetes.pdf.

[25] C. Kramsch , “Research Methods and Approaches in Applied Linguistics: Looking Back and Moving Forward,” AILA Review 27, no. 1 (2014): 30–55, 10.1075/aila.27.02kra.

[26] M. Sandelowski , “Whatever Happened to Qualitative Description?,” Research in Nursing & Health 23, no. 4 (2000): 334–340, 10.1002/1098-240X(200008)23:4<334::AID-NUR9>3.0.CO;2-G. 10940958

[27] M. Sandelowski , “What's in a Name? Qualitative Description Revisited,” Research in Nursing & Health 33, no. 1 (2010): 77–84, 10.1002/nur.20362. 20014004

[28] A. Tong , P. Sainsbury , and J. Craig , “Consolidated Criteria for Reporting Qualitative Research (COREQ): A 32‐Item Checklist for Interviews and Focus Groups,” International Journal for Quality in Health Care 19, no. 6 (2007): 349–357, 10.1093/intqhc/mzm042. 17872937

[29] S. E. Kelly , “Qualitative Interviewing Techniques and Styles,” in The SAGE Handbook of Qualitative Methods in Health Research, ed. I. Bourgeault , R. Dingwall , and R. de Vries (SAGE Publications Ltd, 2010), 307–326, 10.4135/9781446268247.n17.

[30] V. Braun and V. Clarke , “To Saturate or Not to Saturate? Questioning Data Saturation as a Useful Concept for Thematic Analysis and Sample‐Size Rationales,” Qualitative Research in Sport, Exercise and Health 13, no. 2 (2019a): 201–216, 10.1080/2159676X.2019.1704846.

[31] K. Malterud , V. D. Siersma , and A. D. Guassora , “Sample Size in Qualitative Interview Studies: Guided by Information Power,” Qualitative Health Research 26, no. 13 (2016): 1753–1760, 10.1177/1049732315617444. 26613970

[32] V. Braun and V. Clarke , “Using Thematic Analysis in Psychology,” Qualitative Research in Psychology 3, no. 2 (2006): 77–101, 10.1191/1478088706qp063oa.

[33] V. Braun and V. Clarke , “Reflecting on Reflexive Thematic Analysis,” Qualitative Research in Sport, Exercise and Health 11, no. 4 (2019b): 589–597, 10.1080/2159676X.2019.1628806.

[34] Lumivero , *NVivo*. (Version 20). (2020). Lumivero, https://lumivero.com/products/nvivo/.

[35] T. Dunning , J. Speight , and C. Bennett , “Language, the ‘Diabetes Restricted Code/Dialect’, and What It Means for People With Diabetes and Clinicians,” Diabetes Educator 43, no. 1 (2017): 18–26, 10.1177/0145721716683449. 28072923

[36] J. K. Dickinson , S. J. Guzman , and J. S. Wooldridge , “The Emotional Impact of Negative Language in People With Diabetes: A Descriptive Study Using a Semantic Differential Scale,” Science of Diabetes Self‐Management and Care 49, no. 3 (2023): 193–205, 10.1177/26350106231168326. 37052352

[37] A. Cooper , N. Kanumilli , J. Hill , et al., “Language Matters. Addressing the Use of Language in the Care of People With Diabetes: Position Statement of the English Advisory Group,” Diabetic Medicine 35, no. 12 (2018): 1630–1634, 10.1111/dme.13705. 29888553

[38] K. Banasiak , D. Cleary , V. Bajurny , et al., “Language Matters—A Diabetes Canada Consensus Statement,” Canadian Journal of Diabetes 44, no. 5 (2020): 370–373, 10.1016/j.jcjd.2020.05.008. 32616274

[39] J. Speight , T. C. Skinner , T. Dunning , et al., “Our Language Matters: Improving Communication With and About People With Diabetes. A Position Statement by Diabetes Australia,” Diabetes Research and Clinical Practice 173 (2021): 108655, 10.1016/j.diabres.2021.108655. 33422586

[40] C. Hendrieckx , J. A. Halliday , S. Russell‐Green , et al., “Adults With Diabetes Distress Often Want to Talk With Their Health Professionals About It: Findings From an Audit of 4 Australian Specialist Diabetes Clinics,” Canadian Journal of Diabetes 44, no. 6 (2020): 473–480, 10.1016/j.jcjd.2020.02.004. 32360151

[41] E. Litterbach , E. Holmes‐Truscott , F. Pouwer , J. Speight , and C. Hendrieckx , “I Wish My Health Professionals Understood That It's Not Just All About Your HbA1c!'. Qualitative Responses From the Second Diabetes MILES—Australia (MILES‐2) Study,” Diabetic Medicine 37, no. 6 (2020): 971–981, 10.1111/dme.14199. 31802530

[42] M. Peimani , E. Nasli‐Esfahani , and R. Sadeghi , “Patients' Perceptions of Patient–Provider Communication and Diabetes Care: A Systematic Review of Quantitative and Qualitative Studies,” Chronic Illness 16, no. 1 (2020): 3–22, 10.1177/1742395318782378. 29895167

[43] L. Blackwood , J. Gavin , E. Arnott , J. Barnett , C. Dack , and J. Johansen , “#DiabetesOnAPlate: The Everyday Deployment and Contestation of Diabetes Stigma in an Online Setting,” Critical Public Health 33, no. 2 (2022): 160–173, 10.1080/09581596.2022.2077548.

[44] D. Broom and A. Whittaker , “Controlling Diabetes, Controlling Diabetics: Moral Language in the Management of Diabetes Type 2,” Social Science & Medicine (1982) 58, no. 11 (2004): 2371–2382, 10.1016/j.socscimed.2003.09.002. 15047092

[45] E. D. Basinger , M. Farris , and A. L. Delaney , “Investigating the Experience of Diabetes Stigma in Online Forums,” Southern Communication Journal 85, no. 1 (2019): 43–57, 10.1080/1041794X.2019.1655662.

[46] A. Barron , “Irish English and Variational Pragmatics,” in The Oxford Handbook of Irish English, ed. R. Hickey (OUP Oxford, 2023), 400–425, 10.1093/oxfordhb/9780198856153.013.19.

[47] W. Gąsior , “Cultural Scripts and the Speech Act of Opinions in Irish English: A Study Amongst Irish and Polish University Students,” ELOPE: English Language Overseas Perspectives and Enquiries 12, no. 1 (2015): 11–28, 10.4312/elope.12.1.11-28.

[48] E. Vaughan and B. Clancy , “The Pragmatics of Irish English: The Use of English in Ireland Shows Specific Features Which Contribute to Its Unique Profile,” English Today 27, no. 2 (2011): 47–52, 10.1017/S0266078411000204.

[49] E. Vaughan , “Politeness in Irish English,” in The Oxford Handbook of Irish English, ed. R. Hickey (OUP Oxford, 2023), 448–466, 10.1093/oxfordhb/9780198856153.013.21.

[50] M. Ramirez de Arellano , 2014. “The Funny Side of Cross‐Cultural Adaptation: A Grounded Theory Study of the Role of Humour in the Adaptation Process of Spanish Migrants Living in Ireland” (unpublished PhD diss., Dublin City University).

[51] Language Matters: Language and Diabetes , August 22, 2023. NHS, https://www.england.nhs.uk/long-read/language-matters-language-and-diabetes/.

[52] D. S. Dunn and E. E. Andrews , “Person‐First and Identity‐First Language: Developing Psychologists' Cultural Competence Using Disability Language,” American Psychologist 70, no. 3 (2015): 255–264, 10.1037/a0038636. 25642702

[53] C. E. Lloyd , A. Wilson , R. I. G. Holt , C. Whicher , and P. Kar , “Language Matters: A UK Perspective,” Diabetic Medicine 35, no. 12 (2018): 1635–1641, 10.1111/dme.13801. 30103276

[54] M. A. Gernsbacher , “Editorial Perspective: The Use of Person‐First Language in Scholarly Writing May Accentuate Stigma,” Journal of Child Psychology and Psychiatry 58, no. 7 (2017): 859–861, 10.1111/jcpp.12706. 28621486 PMC5545113

[55] C. L. Costigan and M. J. Cox , “Fathers' Participation in Family Research: Is There a Self‐Selection Bias?,” Journal of Family Psychology 15, no. 4 (2001): 706–720, 10.1037/0893-3200.15.4.706. 11770476

[56] K. Dindia and M. Allen , “Sex Differences in Self‐Disclosure: A Meta‐Analysis,” Psychological Bulletin 112, no. 1 (1992): 106–124, 10.1037/0033-2909.112.1.106. 1388280

